# Refeeding the Gut in Anorexia Nervosa: Microbiome, Nutritional Rehabilitation and Dietetic Care

**DOI:** 10.3390/nu18183054

**Published:** 2026-09-18

**Authors:** Panagiota Margioula, Alexandros Nakas, Lilian Adamidou, Eirini Simou, Petros Rafailidis, Theocharis Konstantinidis, Deny Tsakri, Christos Nikolaidis, Despoina Gyriki, Christina Tsigalou, Elisavet Stavropoulou

**Affiliations:** 1 Department of Clinical Nutrition, University General Hospital of Thessaloniki AHEPA, 54636 Thessaloniki, Greece; 2 Diabetes Center, 1st Propaedeutic Department of Internal Medicine, School of Medicine, University General Hospital of Thessaloniki AHEPA, Aristotle University of Thessaloniki, 54636 Thessaloniki, Greece; 3 Independent Researcher, 54638 Thessaloniki, Greece; 4 2nd University Department of Internal Medicine, Department of Infectious Diseases, University General Hospital Alexandroupolis, Democritus University of Thrace, Dragana, 68100 Alexandroupolis, Greece; 5 Laboratory of Microbiology, Department of Medicine, Democritus University of Thrace, Dragana, 68100 Alexandroupolis, Greece; 6 Department of Microbiology, Medical School, National and Kapodistrian University of Athens, 11527 Athens, Greece; 7 2nd Department of Internal Medicine, General Oncology Hospital of Kifissia “Agioi Anargyroi”, 14564 Athens, Greece; 8 Hepatogastroenterology Unit, Academic Department of Internal Medicine, General Oncology Hospital of Kifissia “Agioi Anargyroi”, National and Kapodistrian University of Athens, 14564 Athens, Greece; 9 Laboratory of Hygiene and Environmental Protection, Department of Medicine, Democritus University of Thrace, Dragana, 68100 Alexandroupolis, Greece; 10 Food, Nutrition and Microbiome Program, Laboratory of Hygiene and Environmental Protection, Department of Medicine, Democritus University of Thrace, 68100 Alexandroupolis, Greece; 11 Medical School, University of Nicosia, Athens Campus-UNIC Athens, Elliniko, 16777 Athens, Greece; 12 Infectious Diseases Service, Department of Medicine, Lausanne University Hospital, University of Lausanne, 1015 Lausanne, Switzerland

**Keywords:** anorexia nervosa, gut microbiome, refeeding, nutritional rehabilitation, gastrointestinal tolerance, short-chain fatty acids, probiotics, prebiotics, fermented foods

## Abstract

Anorexia nervosa (AN) is characterized by severe dietary restriction with effects that extend beyond body weight to gastrointestinal function, dietary tolerance and the gut microbiome. Weight restoration remains central to treatment, but studies suggest that microbial composition, microbial metabolites and gastrointestinal symptoms may not recover at the same pace as body mass. This narrative review examines gut microbiome alterations in AN through the clinical context of refeeding and dietetic care. Human and translational studies describe changes in microbial diversity, including shifts in carbohydrate fermenting taxa and their downstream metabolic products such as short-chain fatty acids (including butyrate), mucin-degrading taxa, neuroactive fecal metabolites, immune markers and microbial functional pathways. During refeeding, the substrates available to the gut microbiome are shaped by planned increases in energy intake, the extent to which planned meals are consumed, gastrointestinal tolerance, oral supplement use, constipation, laxative exposure or other medication, dietary variety and the gradual return of fermentable substrates. Current intervention evidence does not support routine use of probiotics, prebiotics or synbiotics for weight restoration, eating-disorder psychopathology or targeted microbiome modulation. Fiber-containing foods, fermented foods and dietary diversity may be relevant to dietary substrate exposure and gastrointestinal tolerance, but their use during AN rehabilitation should be framed as pragmatic dietetic care rather than as established microbiome-specific treatment. Their introduction should remain secondary to adequate energy provision, tolerance, normalization of eating patterns and avoidance of reinforcing restrictive behaviors. Dietitians can translate microbiome-related knowledge into cautious nutritional decision-making while ensuring that patient care remains centered on rehabilitation, safety and dietary adequacy.

## 1. Introduction

Anorexia nervosa (AN) is a psychiatric disorder characterized by restrictive eating, fear of weight gain and disturbance in body image [1]. Although its point prevalence is relatively low, lifetime prevalence has been estimated at up to 4% in females and 0.3% in males [2]. However, its clinical course cannot be understood only through psychiatric symptoms or body weight. Prolonged energy restriction affects almost every system involved in nutritional homeostasis, including endocrine function [3], gastrointestinal motility [4], immune activity [5], body composition [6] and energy metabolism [7]. Therefore, treatment aims not only to reverse low weight, but also to restore nutritional status, physical function, and the patient’s capacity to tolerate and maintain adequate food intake over time [8].

Weight restoration remains a main objective of AN treatment and is strongly associated with medical stabilization and improved prognosis [8]. Nevertheless, weight gain does not always mean complete biological recovery. Long-term follow-up data show that recovery is often prolonged [9], with one review reporting persistent symptoms or disability in approximately 40% of patients after 10 years of medical care [10]. Many patients continue to report gastrointestinal symptoms during and after nutritional rehabilitation, including early satiety, bloating, constipation, abdominal discomfort and altered hunger or fullness cues [4,11]. These symptoms may make it harder for patients to consume planned meals, increase anxiety around eating and make the refeeding process more difficult [11,12]. Therefore, recovery should be evaluated not only through body mass index or short-term weight gain, but also through the restoration of gastrointestinal function, dietary variety and metabolic stability [8,13].

The gut microbiome is of particular interest in this context because it is directly shaped by dietary intake, energy availability and the composition of the diet [14]. In AN, prolonged restriction changes the substrates available to intestinal microorganisms and may favor microbial communities adapted to a low-energy and substrate-limited intestinal environment [15]. Studies in patients with AN have reported alterations in microbial diversity and composition, including changes in butyrate-producing bacteria, mucin-degrading taxa and microbial metabolites such as short-chain fatty acids (SCFAs) [16,17,18,19]. Although findings are not fully consistent across studies, human studies in AN suggest that the disorder is associated with changes in the gut microbiota that may be relevant to microbial fermentation, energy metabolism and gut–brain communication. Translational evidence supports biological plausibility, but does not provide direct clinical guidance for nutritional rehabilitation [16,17,20].

Refeeding therefore represents a critical period and a unique opportunity to explore the relationship between dietary changes and the gut microbiota [21]. Food variety [22,23], enteral or oral nutritional support [24,25] and medication use [26] may all influence the intestinal environment. In AN, these factors occur within refeeding protocols in which the pace of energy increase is clinically important [27], although direct evidence linking caloric advancement to microbiome recovery remains limited [17]. At the same time, gastrointestinal tolerance often determines whether nutritional targets can be achieved in practice [28]. From this perspective, refeeding may also be considered as a process of rebuilding the nutritional and microbial conditions required for sustained recovery [17,29,30].

Several mechanisms have been proposed to explain how gut microbiome alterations could be linked to symptoms and recovery in AN. Microbial metabolites, especially SCFAs, may influence intestinal barrier integrity, immune signaling and energy metabolism [31,32]. Other pathways involve gut-derived hormones, bile acids, tryptophan metabolism, vagal signaling and neuroactive compounds related to appetite, mood and satiety [33,34]. Molecular mimicry involving bacterial proteins such as caseinolytic peptidase B (ClpB) and appetite-regulating peptides has also been discussed as a possible mechanism, although its clinical significance remains under investigation [35]. Although causality between gut microbiome alterations and AN cannot currently be established, these mechanisms suggest that the gut microbiome may participate in symptom persistence and the response to refeeding or influence the ability to achieve stable nutritional recovery in some patients [17,20].

Interest in microbiome-related dietary strategies has therefore increased, particularly in relation to probiotics and prebiotics as possible adjuncts to nutritional rehabilitation in AN [36]. Dietary fiber [37], fermented foods [38] and broader dietary diversification [39] may also be relevant because they can influence microbial diversity, fermentation patterns and immune-related markers in human studies, although most of this evidence comes from populations other than AN. In AN, the evidence remains limited and should be interpreted carefully. In addition, strategies that are generally considered beneficial for gut health may not be immediately tolerated during early refeeding, when gastrointestinal symptoms, altered perceptions of appetite, anxiety around eating, and limited dietary variety are common [28,40]. Thus, microbiome-targeted nutritional strategies should not be presented as a replacement for established treatment, but rather as a potential adjunctive approach that requires careful clinical judgment and further research.

These issues are directly relevant to dietetic care and clinical nutrition practice. Clinical nutrition professionals are involved in assessing nutritional status, planning energy progression, monitoring refeeding risk, adapting meal plans, supporting dietary variety and managing gastrointestinal symptoms during recovery [41]. They are also well placed to evaluate when and how fiber, fermented foods, prebiotics or probiotics could be introduced safely, without reinforcing restrictive eating patterns or unnecessary food rules. In a multidisciplinary setting, they can help translate microbiome-related evidence into practical decisions, while avoiding overinterpretation of findings that are still preliminary.

Existing reviews have described gut microbiome alterations in AN and their possible links with the gut–brain axis [20,42,43]. However, less attention has been given to how these findings should be interpreted during nutritional rehabilitation, when energy intake, food variety, gastrointestinal tolerance and symptom-related food avoidance are changing together. Hence, the present narrative review examines gut microbiome alterations in AN through the practical context of refeeding and dietetic care, with emphasis on nutritional recovery, microbiome-derived metabolites, gastrointestinal function and the safe use of dietary strategies relevant to the gut microbiome.

## 2. Materials and Methods

This is a narrative literature review integrating evidence from selected studies identified through searches of PubMed, Scopus, and Google Scholar, covering articles from database inception to June 2026, with the final search completed in June 2026. The following search terms were used alone or in combination for the literature review: “anorexia nervosa”, “eating disorder”, “gut microbiome”, “gut microbiota”, “dysbiosis”, “microbial metabolites”, “short-chain fatty acids”, “SCFAs”, “butyrate”, “refeeding”, “renourishment”, “nutritional rehabilitation”, “weight restoration”, “gastrointestinal symptoms”, “constipation”, “dietary variety”, “dietary fiber”, “fermented foods”, “probiotics”, “prebiotics”, “synbiotics”, “dietitian” and “dietetic care”. Boolean operators (AND, OR) were used to refine and combine search terms. Articles were selected based on their relevance to the emerging role of the gut microbiome in AN and its potential implications for nutritional rehabilitation and dietetic care. Non-English publications, case reports, conference abstracts, commentaries and editorials were excluded.

Studies were selected when they addressed gut microbiome composition, microbial metabolites, gastrointestinal symptoms, refeeding, nutritional rehabilitation, dietary variety, fiber-containing foods, fermented foods, probiotics, prebiotics, synbiotics or dietetic care in relation to anorexia nervosa. Priority was given to systematic reviews and meta-analyses when available, followed by human studies in anorexia nervosa, particularly longitudinal, interventional and multi-omics studies. Evidence from healthy populations, other clinical populations and animal or translational models was included when it helped interpret mechanisms or identify areas for future research, but it was not treated as equivalent to AN-specific clinical evidence. Because this was a narrative review, no meta-analysis was performed.

To make the strength and applicability of the evidence clearer, we considered the type of evidence and its direct relevance to AN rehabilitation. Review articles, systematic reviews and meta-analyses were used where available, particularly when discussing recurring findings and areas of uncertainty. AN-specific human studies were considered most relevant to clinical interpretation, especially longitudinal, interventional and multi-omics studies. Smaller cross-sectional studies were interpreted cautiously and mainly in relation to findings reported elsewhere. Evidence from healthy adults or other clinical populations was used only when AN-specific data were limited and when the topic was directly relevant to dietary substrates, gastrointestinal tolerance, SCFA production, fermented foods, fiber-containing foods or probiotic/prebiotic interventions. Animal and microbiome-transfer studies were discussed as evidence of biological plausibility, not as a basis for dietetic recommendations. This distinction was used to separate established principles of nutritional rehabilitation from microbiome-related hypotheses and adjunctive strategies that require further study.

## 3. Weight Restoration and Gastrointestinal, Dietary and Microbiome-Related Outcomes

Weight restoration remains a central goal of AN treatment because it is closely linked to medical stabilization and short-term clinical improvement [8,13]. Still, body weight alone cannot describe the broader process of biological recovery. In this review, the term “gut-related recovery” is used only as a shorthand for several distinct domains: gastrointestinal tolerance, dietary variety, gut microbiome composition and microbial metabolite profiles. It is not used as a validated clinical endpoint, and these domains should not be assumed to improve at the same pace. Gastrointestinal complaints may persist despite weight gain [17,28], dietary variety may remain limited during early recovery [13,44], and longitudinal studies show that the gut microbiome may continue to differ from that of healthy individuals after renourishment or short-term weight restoration [17,29,45,46]. Similar findings have been reported for microbial metabolites, including SCFAs and neurotransmitter-related fecal metabolites, which may not fully recover during hospitalization or early weight restoration [17,45,47]. The available evidence therefore points to a mismatch between weight gain and gut-related recovery during nutritional rehabilitation.

Mack et al. provided one of the first longitudinal indications that weight gain does not necessarily restore gut microbiome composition or microbial metabolite patterns in AN. In their study, fecal microbiome, SCFAs, dietary intake and gastrointestinal complaints were assessed in patients with AN before and after inpatient weight gain and compared with normal-weight controls [17]. The authors reported microbial perturbations during the underweight state, including changes in mucin-degrading bacteria, *Clostridium* clusters and the butyrate-producing genus *Roseburia*. After weight gain, microbial richness increased, but the fecal microbiome profile, branched-chain fatty acid pattern and several gastrointestinal complaints did not normalize. Thus, the improvement in body weight was not matched by a similar recovery of fecal microbiome composition, microbial fermentation profiles or gastrointestinal complaints.

Kleiman et al. reached a similar conclusion in a smaller inpatient cohort. They examined the intestinal microbiome in acute anorexia nervosa and again during inpatient renourishment [29]. Although the microbiome changed during treatment, alpha diversity remained lower than in healthy controls at both admission and discharge. The study also reported associations between microbial measures and depression, anxiety and eating-disorder psychopathology, suggesting that microbiome changes during renourishment may be relevant beyond the gastrointestinal tract. Although the sample size was limited, the study was important because it followed the same patients across treatment rather than relying only on cross-sectional comparisons.

Later studies examined this mismatch in more detail by combining follow-up sampling with microbiome and metabolite analyses. Monteleone et al. examined women with anorexia nervosa in the underweight phase and after short-term weight restoration, comparing 21 underweight patients, 16 weight-restored patients and 20 healthy women [45]. Compared with controls, patients showed decreased intra-individual bacterial richness, an increased *Bacteroidota*-to-*Bacillota* ratio (reported as a *Bacteroidetes*-to-*Firmicutes* ratio in the original study) and changes in bacterial abundance at several taxonomic levels. These alterations were observed both in the underweight and weight-restored conditions. The authors also integrated microbiome data with 49 fecal metabolites and found that bacteria–metabolite relationships differed across groups, with divergent patterns in acutely ill patients compared with weight-restored patients and controls. Importantly, the authors noted that short-term BMI normalization should not be interpreted as stable weight recovery or full recovery from AN.

Similar findings have been reported in adolescents. Schulz et al. studied 19 female adolescents with anorexia nervosa at admission and after short-term weight recovery, comparing them with 20 healthy controls [46]. Nutritional therapy started at 1200 kcal/day and increased by 200 kcal every second day until weight gain reached 500–1000 g/week and the 20th–25th age-adapted BMI percentile. Alpha diversity increased after short-term weight recovery, but beta diversity still showed clear differences from healthy controls before and after weight gain. The authors also reported reduced *Romboutsia* and *Enterobacteriaceae*-related taxa at both time points, while unclassified *Lachnospiraceae* increased at discharge. The authors concluded that gut microbiome alterations in adolescent anorexia nervosa did not normalize with short-term weight restoration.

Prochazkova et al. extended this question by examining bacterial composition, mycobiome, fecal neurotransmitters and SCFAs in patients with severe anorexia nervosa before and after hospitalization [47]. Patients had a median BMI of 14.4 kg/m^2^ before treatment and 17.1 kg/m^2^ after hospitalization, with significant improvement in BMI and eating-disorder psychopathology. However, several microbial and metabolite abnormalities persisted. The study reported signs of core microbiome depletion, with underrepresentation of taxa such as *Faecalibacterium*, *Agathobacter*, *Bacteroides*, *Blautia* and *Lachnospira*, and lower fecal levels of serotonin, gamma-aminobutyric acid (GABA), dopamine, butyrate and acetate compared with controls. Although patients showed partial nutritional recovery, SCFA profiles, neurotransmitter-related fecal metabolites and microbial community composition did not change substantially during hospitalization.

Microbial composition, metabolite production and gastrointestinal symptoms may remain altered even when BMI improves. For this reason, microbiome-related recovery should not be judged only through diversity indices, but also through microbial composition, fermentation patterns and fecal metabolites. This is particularly relevant for SCFAs and branched-chain fatty acids, because they reflect microbial fermentation of carbohydrates and proteins and may be influenced by dietary intake, gut transit time and microbial community structure [17,45,47]. In this context, the term “recovery” should be used carefully. Increased BMI, improved psychometric scores and reduced acute medical risk may occur while microbial metabolites or gastrointestinal symptoms remain altered.

The clinical expression of this incomplete recovery is often seen through gastrointestinal symptoms. During nutritional rehabilitation, patients may continue to experience abdominal fullness, bloating, constipation, abdominal pain, bowel noises or incomplete evacuation [17,28]. These symptoms can interfere with the consumption of planned meals, reinforce fear of fullness and make the gradual reintroduction of foods more difficult. For this reason, gastrointestinal tolerance should be considered alongside weight gain when evaluating nutritional rehabilitation. A patient may be gaining weight, but still eating a narrow range of foods, avoiding fiber, relying heavily on oral supplements or struggling with symptoms that limit normal eating patterns.

The evidence should still be interpreted with caution. The available longitudinal studies are few, sample sizes are small, and the studies differ in age group, illness duration, treatment setting, discharge BMI, medication use, sequencing methods and dietary exposure during treatment. Reviews focusing on the gut microbiome in anorexia nervosa and nutritional rehabilitation have also noted that longitudinal evidence remains limited and that microbiome findings are not fully consistent across studies [30,43]. Therefore, current data do not prove that persistent dysbiosis causes poor recovery or relapse. A more cautious interpretation is that body weight, gastrointestinal symptoms, microbial composition and microbial metabolites may not recover at the same pace.

## 4. Nutrition-Relevant Gut Microbiome Alterations in Anorexia Nervosa

Human studies in AN have repeatedly reported disturbances in the gut microbiome, although the reported changes vary across cohorts, illness stages and analytical methods. Differences have been described in microbial diversity, taxonomic composition, metabolite profiles and functional pathways [20,30,43]. Part of this variability is expected, given differences in age, illness duration, illness subtype, recent intake, medication exposure, gastrointestinal symptoms and stage of nutritional rehabilitation. For this reason, the most useful way to interpret the literature is to examine which alterations may be relevant to dietary restriction, substrate availability, fermentation and nutritional recovery. The main findings are summarized in Table 1.

Microbial diversity is often considered a marker of gut health, but in AN it should be interpreted carefully. Lower alpha diversity has been reported in the study by Kleiman et al. [29]. However, diversity findings are not consistent across all studies or all diversity indices [20]. In Prochazkova et al., for example, the only increased alpha diversity parameter before treatment was the Chao1 index, which the authors interpreted as reflecting greater interindividual variation rather than simple microbial enrichment [47]. Beta diversity findings, which reflect differences in community composition between groups, may be more informative in some cohorts, especially when they remain different from controls after short-term weight gain [46]. Longer adolescent follow-up data also suggest that microbiome differences may diminish with weight recovery, energy intake and hormonal recovery, although this does not mean that early inpatient weight gain is equivalent to full gut-related recovery [48]. Diversity therefore may indicate that the gut microbiome differs between patients and controls, but it is not sufficient on its own to judge nutritional or microbial recovery.

A more directly nutrition-related pattern concerns bacteria involved in carbohydrate fermentation and short-chain fatty acid production. Several studies have reported lower or altered abundance of taxa such as *Roseburia*, *Faecalibacterium*, *Lachnospira*, *Blautia* and *Agathobacter* [16,17,18,47]. These bacteria are relevant because many members of these groups participate in the fermentation of non-digestible carbohydrates and the production of metabolites such as butyrate [52,53,54,55]. In AN, prolonged restriction, low dietary variety and reduced intake of fermentable substrates may alter the conditions that support these organisms [39,52,54]. Therefore, the reduction in individual bacterial taxa should not be interpreted as an explanation for AN pathogenesis. A more plausible interpretation is that prolonged dietary restriction changes the substrates reaching the colon, shaping both microbial composition and metabolic activity.

The question of substrate use can also be examined at the functional level. In a shotgun metagenomic study from two geographically distinct eating disorder units, Fouladi et al. examined fecal samples from patients with AN before and after inpatient treatment. Although patients gained weight during treatment, their post treatment microbiomes remained distinct from t those of controls without eating disorders [50]. Before treatment, the AN microbiome also showed differences in metagenomic pathway profiles, with greater representation of fermentation pathways and lower carbohydrate degradation capacity compared with controls. In this context, the relevance of the microbiome is not limited to which bacteria are more or less abundant, but also concerns how the gut community handles dietary substrates during illness and refeeding [50,54,55].

Another recurring finding involves mucin-degrading bacteria, particularly *Akkermansia muciniphila*. Some studies [17,49] and reviews [30,43] describe increased mucin-degrading organisms in AN, including *Akkermansia*, and discuss this as a possible adaptation to low dietary substrate availability. This interpretation should remain cautious. *Akkermansia muciniphila* should not be interpreted as harmful simply because it is increased in some AN cohorts. In AN, its meaning may be different because the gut is exposed to prolonged restriction, altered transit and limited fermentable substrates. A higher reliance on mucus-related substrates could therefore represent a microbial response to dietary restriction rather than a primary abnormality.

The same diet dependent pattern is also seen in fecal metabolites. Lower fecal concentrations of selected SCFAs, including acetate, propionate, butyrate or total SCFAs, have been reported in some AN cohorts [18,19,47]. Mack et al. also reported altered branched-chain fatty acid profiles after weight gain [17], while Monteleone et al. found altered bacteria metabolite relationships in underweight and short-term weight-restored patients [45]. Fecal metabolite levels depend on microbial production, absorption, stool transit and recent intake, including the availability of fermentable carbohydrates and protein substrates [54,56,57,58,59,60]. Their value lies in showing how the gut microbiome is functioning within the nutritional context of restriction and refeeding, rather than in serving as standalone indicators of recovery [57,58].

Some studies also extend the discussion to gut barrier function and immune signaling. Reduced butyrate production may be relevant because butyrate contributes to epithelial barrier function and immune regulation, although direct human evidence for barrier disruption in AN remains limited [31,42,53]. Findings related to immune markers in AN should also be interpreted carefully. In adolescents with AN, Käver et al. found lower IL 1β and IL 6 during the acute phase, normalization of IL 1β levels after weight recovery, no significant difference in TNF α and persistently elevated IL 15, with associations between cytokines, clinical variables and gut microbiome [51]. The cytokine results are mixed at the level of individual markers. They are better discussed in relation to nutritional state, illness duration and recovery stage than as evidence of one inflammatory phenotype.

For nutritional practice, microbiome evidence becomes meaningful only when it is translated back into the context of refeeding. To date, the aim is not to correct individual bacterial taxa or prescribe interventions according to microbial markers, as current evidence does not yet support their use as clinical decision-making tools [36,41,43]. Instead, microbiome-related insights should complement established principles of nutritional rehabilitation. The more relevant issue is how nutritional rehabilitation changes the pattern of intestinal substrate exposure. As energy intake increases and food variety is restored, the gut is exposed again to substrates that may have been scarce, including fermentable carbohydrates, fiber-containing foods and protein residues. The evidence therefore places refeeding beyond weight gain alone, as a process that also involves nutritional adequacy, gastrointestinal tolerance and the gradual restoration of flexible eating.

## 5. Refeeding, Gastrointestinal Tolerance and Microbial Adaptation

Studies of refeeding in AN usually focus on how nutritional intake is advanced, whether weight is restored safely and whether medical stability is achieved [27,61,62], whereas microbiome studies usually focus on fecal samples, microbial composition and metabolite profiles [17,29,45,47,50]. Less attention is given to the intake that is actually completed, tolerated, digested and delivered to the intestine over time. Sustained substrate exposure for the gut microbiome depends on what is repeatedly consumed and tolerated, not only on what is planned. During early nutritional rehabilitation, patients may be increasing intake while still experiencing postprandial fullness, bloating, abdominal distension, constipation, abdominal pain or anxiety around gut sensations [28,40,63,64]. Gastrointestinal tolerance is therefore one of the conditions that determines whether energy targets, food variety, fiber-containing foods and fermentable substrates can be restored in practice.

Several gastrointestinal symptoms in AN have physiological correlates, although they are often intensified by anxiety and fear of eating. Delayed gastric emptying, impaired gastric accommodation and delayed intestinal transit have all been described in AN, and reviews emphasize that gastrointestinal complaints can complicate treatment, particularly when they are interpreted by patients as evidence that eating is harmful or physically intolerable [63,64]. In a prospective study, Waldholtz and Andersen found that multiple gastrointestinal symptoms were present at admission and that most improved during refeeding, despite substantial increases in caloric intake [65]. The improvement occurred while intake was being advanced, which is important when gastrointestinal discomfort is used to question the pace of nutritional rehabilitation. Perez et al. also studied adolescent females with AN and showed that impaired gastric accommodation improved after nutritional rehabilitation, with fewer patients meeting criteria for functional gastrointestinal disorders after treatment, while anxiety symptoms persisted [66]. This separates improvement in gastrointestinal function from full resolution of the anxiety attached to eating and postprandial sensations.

A similar pattern appears during inpatient treatment. In the study by Riedlinger et al., abdominal pain and constipation improved over time, whereas symptoms related to indigestion and abdominal distension were more resistant during the observed treatment period [28]. Heruc et al. reported a comparable finding in adolescent females with AN: after two weeks of refeeding, peptide YY and appetite perceptions improved, but bloating, elevated ghrelin and postprandial anxiety persisted [40]. A patient may therefore gain weight or become medically more stable while meals remain difficult to complete. Fullness, visible distension, bowel noises or constipation may slow eating, reinforce avoidance of specific foods and make dietary expansion feel physically unsafe. These symptoms matter because they affect how regularly food is consumed, how much variety is tolerated and how consistently fermentable substrates reach the intestine.

Faster refeeding has answered one clinical question more clearly than it has answered a microbiome-related one. The StRONG trial showed that higher-calorie refeeding in hospitalized adolescents and young adults restored medical stability earlier than a lower-calorie approach, without an increase in electrolyte abnormalities under appropriate monitoring [27]. These results support carefully monitored advancement of intake as part of medical stabilization. They do not show that gut microbiome composition, microbial metabolism or gastrointestinal tolerance recover at the same pace. The same energy target can be reached through different meal structures, different proportions of oral supplements, different levels of food variety and different amounts of fiber or fermentable carbohydrate. As a result, two patients following similar increases in intake may expose the gut to very different substrates. Refeeding risk remains a separate clinical concern, particularly in severe malnutrition, and requires electrolyte monitoring and medical oversight according to established risk frameworks [67]. For the gut, the relevant distinction is between planned intake, completed and tolerated intake, and the substrates that actually enter the gastrointestinal tract.

**Figure 1:**
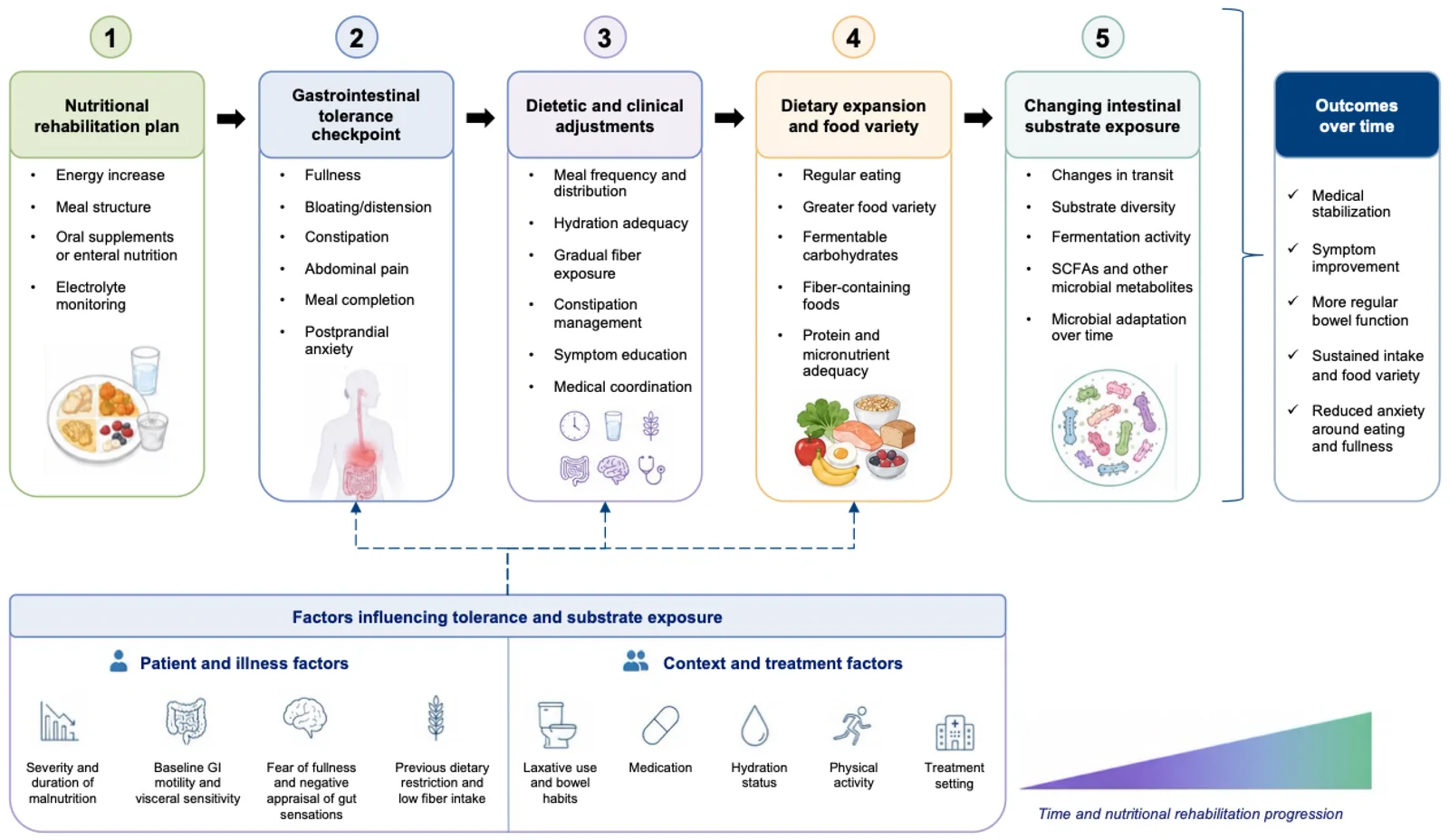
Gastrointestinal tolerance as the link between nutritional rehabilitation and intestinal substrate exposure in anorexia nervosa. During rehabilitation, increased energy intake affects intestinal substrate exposure through regular consumption of planned meals, tolerance of postprandial sensations and gradual expansion of food variety. Fullness, bloating, constipation, abdominal pain, stool changes and anxiety around eating can influence this progression. As intake becomes more regular and varied, fermentable carbohydrates, fiber-containing foods and protein residues may contribute to changes in transit, fermentation, SCFA production and microbial adaptation over time. Abbreviations: SCFAs, short-chain fatty acids.

Constipation has particular relevance during refeeding because it affects both meal tolerance and the delivery of fermentable substrates to the colon. In AN, constipation can result from low intake, delayed whole-gut transit, reduced stool bulk, inadequate hydration, reduced activity, fiber restriction, laxative misuse or laxative withdrawal [64,68]. A recent scoping review on constipation management in eating disorders described constipation as a nutrition-impact symptom and noted that evidence for its management in AN and bulimia nervosa remains limited and heterogeneous [69]. Fiber and fermentable carbohydrates are important for fermentation and stool characteristics [39], but their timing during nutritional rehabilitation matters. When fiber is increased before overall intake is adequate, or advanced too quickly, bloating, fullness and fear of abdominal distension may worsen. Nutritional adequacy and meal regularity should be established before gradual expansion of fiber-containing and fermentable foods according to stool pattern, hydration, abdominal symptoms, the patient’s ability to consume planned meals and tolerance of postprandial sensations, as part of the route from nutritional rehabilitation to changing substrate exposure in the gut (Figure 1).

Nutritional adequacy and meal regularity should be established before gradual expansion of fiber-containing and fermentable foods according to stool pattern, hydration, abdominal symptoms, the patient’s ability to consume planned meals and tolerance of postprandial sensations. Figure 1 summarizes this sequence as a practical dietetic link between nutritional rehabilitation, gastrointestinal tolerance and changing substrate exposure in the gut.

Microbial changes during refeeding are better linked to the pattern of substrate delivery than to weight gain alone. Fecal microbiome and metabolite profiles obtained during treatment reflect the cumulative effects of meals that were actually completed and tolerated, including fermentable carbohydrates, dietary fibers and protein residues reaching the colon. When restriction has reduced meal volume, dietary variety and fermentable substrate intake, nutritional rehabilitation changes the amount, timing and composition of material entering the gut, not only total energy intake. Persistent differences from controls after short-term weight gain can be consistent with limited time, incomplete dietary expansion, ongoing gastrointestinal symptoms and heterogeneous substrate delivery [17,46]. Metagenomic findings of altered microbial functional capacity in AN add to this interpretation, because they indicate differences not only in which microbes are present, but also in how available substrates can be metabolized [50]. Accordingly, microbiome recovery is likely to depend on sustained, tolerated and nutritionally varied intake over time rather than on weight gain alone.

Sustained, tolerated and varied intake also defines the limits of interpretation of current microbiome research. Gastrointestinal symptoms can guide pacing, education and bowel management, but they should not be used to justify avoidant restriction when nutritional rehabilitation is medically necessary [8,13,64]. Similar caution is needed when microbiome-related strategies are considered during AN rehabilitation. Fiber-containing foods and fermentable substrates are relevant to recovery, but rapid expansion may worsen bloating, fullness or constipation and make planned meals harder to consume when tolerance is still limited [39,52,53,54]. Refeeding must restore adequacy, regularity and variety while tolerance is rebuilt. At present, the microbiome is best understood as part of the nutritional environment created during rehabilitation, rather than as a direct target for manipulation of selected taxa in routine AN care [36,43].

## 6. Microbiome-Related Pathways in Appetite, Mood and Recovery

Evidence linking the gut microbiome with appetite, mood and recovery in AN remains suggestive rather than settled. Human studies in AN report changes in microbial composition, fecal metabolites and immune markers. Animal-transfer experiments are considered separately because they test biological plausibility under controlled experimental conditions, rather than clinical applicability to nutritional rehabilitation. Their relevance is greatest during refeeding, when intake, gastrointestinal symptoms and substrate availability are changing at the same time. They do not show that AN is caused by dysbiosis, or that routine care should aim to manipulate selected taxa or metabolites [36,43,70].

### 6.1. Fermentation Products and Appetite-Related Signaling

SCFAs provide one of the clearest links between dietary substrate availability, microbial metabolism and host signaling. Acetate, propionate, and butyrate mainly come from bacterial fermentation of non-digestible carbohydrates, resistant starch, and dietary fibers. Their effects extend beyond the colon, impacting epithelial metabolism, mucus production, barrier function, immune responses, enteroendocrine activity, and microbiome–gut–brain interactions [31,32,39,71]. In AN, this pathway is closely tied to substrate availability, since dietary restriction changes the amount and type of fermentable material reaching the colon, while refeeding gradually modifies the substrate environment available to microbial communities.

Human studies in AN support alterations in microbial fermentation. However, these changes should not be interpreted as biomarkers of recovery. Prochazkova et al. reported lower fecal acetate and butyrate in severe AN, with minimal change after partial renourishment [47]. Monteleone et al. identified altered relationships between gut bacteria and fecal metabolites in underweight and weight-restored patients, suggesting that BMI normalization alone does not restore the bacteria–metabolite links seen in controls [45]. During rehabilitation, microbial fermentation depends on more than increased intake. The amount of food actually consumed, food variety, transit time and gradual exposure to fiber-containing and fermentable foods all affect the substrates that reach the colon [39,53,54]. Fecal SCFA levels are hard to interpret because they are influenced by production, absorption, stool transit, sample handling, and recent intake [57,58,60]. Their interpretation is most meaningful when combined with dietary exposure and clinical tolerance rather than as standalone markers of microbial recovery.

### 6.2. Neuroactive and Tryptophan-Related Metabolism

Neuroactive metabolites raise a separate issue from fermentation. The key question is whether intestinal metabolism contributes to appetite perception, visceral sensations and mood-related symptoms during recovery, while avoiding interpretation of fecal metabolites as direct indicators of brain neurotransmission. Gut–brain communication includes enteric and vagal signaling, immune mediators, enteroendocrine hormones, microbial metabolites and central processing of visceral signals [33,34,72]. These pathways are especially relevant to AN, as feelings of fullness, nausea, anxiety related to eating, and changes in appetite perception can hinder nutritional recovery.

Tryptophan metabolism is central to this discussion. Gut bacteria can influence the balance among serotonin, kynurenine and indole-related pathways, which are linked to gastrointestinal function, epithelial signaling, immune regulation and central nervous system biology [73]. In AN, Prochazkova et al. provide one of the few direct evidence from fecal samples, showing lower levels of serotonin, GABA, and dopamine in severe cases [47]. This finding should be interpreted as evidence of altered intestinal metabolism, not as a marker of central neurotransmission. Fecal levels of serotonin, GABA, or dopamine do not reflect central neurotransmission and cannot establish a microbial cause of anxiety, depression, satiety, or food avoidance. Instead, they suggest altered intestinal neuroactive metabolism in a disorder where appetite perception and psychological symptoms are clinically prominent.

### 6.3. Barrier, Mucus and Immune Signaling

Barrier and immune pathways are also relevant, but the human evidence in AN remains uneven. Butyrate contributes to epithelial energy metabolism and barrier-related signaling in the broader gastrointestinal literature [31]. During prolonged dietary restriction, reduced availability of fermentable substrates could alter the metabolites available to epithelial cells and may increase the relative importance of host-derived substrates such as mucus. This is consistent with earlier observations of changes in mucin-degrading bacteria and with review-level hypotheses linking AN-related dysbiosis to intestinal permeability, mucosal barrier function and immune signaling [17,42,43].

The immune findings are difficult to reduce to a single inflammatory explanation. Meta-analyses have shown altered cytokine levels in AN, including higher TNF-α and IL-6 in some pooled analyses [74,75]. More recent studies, however, reveal a less uniform pattern. In adolescents with AN, Käver et al. found reduced levels of IL-1β and IL-6 during the acute phase, with IL-1β levels normalizing after weight restoration. They reported no significant differences in TNF-α and persistently elevated IL-15, with links between cytokines, clinical factors, and gut microbiome [51]. A newer meta-analysis also indicated higher IL-6 and IL-15 and lower IL-7 in AN, while other cytokines showed no differences after accounting for outliers [76]. Overall, these findings suggest immune dysregulation during illness and recovery but do not support the characterization of AN as a uniformly pro-inflammatory state. Barrier and immune signaling remain possible routes through which malnutrition, gastrointestinal symptoms, microbial metabolites and recovery stage may interact, but direct evidence linking barrier dysfunction to clinical recovery in AN remains limited.

### 6.4. ClpB/α-MSH Molecular Mimicry

ClpB/α-MSH molecular mimicry is among the most specific microbiome-related hypotheses. Tennoune et al. identified bacterial caseinolytic peptidase B as a conformational mimetic of α-MSH, showing that immunizing mice with ClpB generated antibodies cross-reactive with α-MSH, affecting food intake, body weight, anxiety behaviors, and melanocortin receptor signaling [34]. Human data on eating disorders from the same study indicated altered profiles of anti-ClpB antibodies that cross-react with α-MSH [35]. Later, Lucas et al. found increased α-MSH-reactive IgG in patients with anorexia and bulimia, and these α-MSH/IgG complexes influenced melanocortin-4 receptor signaling in experimental systems [77]. Because this evidence includes experimental models and mixed eating-disorder samples, it should be interpreted as a mechanistic hypothesis rather than as direct evidence for AN treatment.

The value of this pathway lies in its specificity, since it proposes a defined molecular connection between gut bacteria, adaptive immunity and satiety signaling. It goes beyond a simple claim that “dysbiosis affects appetite.” Fetissov and Hökfelt integrated this mechanism into a broader hypothesis involving altered communication between gut microbiome, adaptive immunity, and melanocortin pathways in eating disorders [78]. Recent targeted proteomic studies have also identified α-MSH-like proteins from enterobacteria, such as ClpB, in fecal samples from AN patients, although these findings were preliminary and based on small samples [79]. Caution is needed in clinical interpretation. The pathway has not been shown to explain appetite dysregulation, symptom persistence or recovery in AN at the individual patient level. It is best viewed as a specific biological hypothesis that might influence appetite-related signaling in some situations, rather than an established mechanism or treatment option.

### 6.5. Translational Evidence from Microbiome-Transfer Studies

Microbiome-transfer studies come closest to establishing causality, although their results are inconsistent. Fan et al. used human metagenomics and metabolomics combined with fecal microbiota transplantation into germ-free mice on energy-restricted diets. These mice, receiving microbiota from AN patients, gained less weight and exhibited changes in hypothalamic and adipose tissue gene expression related to energy metabolism and eating behavior [16]. Similarly, Hata et al. found that gut microbiota transfer from patients with AN reduced weight gain and altered behavioral performance in female mice [80]. These findings strengthen the biological plausibility of microbiome involvement by moving beyond human associations to transferable effects in controlled models.

Other studies have not replicated the same effect. Glenny et al. observed that gut microbial communities from patients with AN did not affect body weight in germ-free mice recipients [81]. Kooij et al. found no evidence that fecal microbiota transplants from AN patients changed cognitive flexibility, anxiety, or dopamine-related endpoints in rats, although the study also highlighted limited microbiota overlap between donors and recipients after transplantation [82]. More recent research by Maschek et al., involving antibiotic-treated mice, showed reduced food intake after transplantation from AN donors, along with changes in gut microbiota composition and appetite-related hormones, including PYY and leptin, but no effect on body weight during the first transplant phase [83]. The mixed findings are useful because they prevent a simple causal reading of FMT experiments. They should therefore inform mechanistic interpretation rather than routine dietetic decisions in AN care.

The main studies supporting these proposed pathways are presented in Table 2.

These studies suggest that the microbiome may contribute to AN pathophysiology, although translating from experimental models to human clinical care remains challenging. AN-associated microbiome can influence weight gain, food intake, behavior or appetite-related signaling in some experimental settings, but these effects are inconsistent across models and cannot yet be translated into a clinical treatment claim. For human recovery, the more defensible conclusion remains that microbiome-related pathways likely interact with the nutritional conditions created during rehabilitation. Sustained intake, dietary variety, exposure to fermentable substrates, bowel function and gastrointestinal tolerance remain the clinically accessible variables that shape that environment. This provides a rationale for considering microbiome-directed adjunctive strategies, such as probiotics, prebiotics and fermented foods, but only as supportive approaches that should be evaluated within the broader goals of nutritional rehabilitation, medical stability and psychological recovery.

## 7. Dietary Strategies Relevant to the Gut Microbiome During Nutritional Rehabilitation

### 7.1. Probiotics, Prebiotics and Synbiotics

Intervention studies in AN have evaluated several microbiome-related strategies, including live-culture fermented dairy products, strain-defined probiotics and, less frequently, prebiotic or synbiotic approaches. Probiotics are live microorganisms that confer a health benefit when administered in adequate amounts. Prebiotics are selectively utilized substrates that confer a host benefit, and synbiotics combine both components in a defined way [84,85,86]. Early intervention studies in AN were based mainly on live-culture dairy products provided during nutritional rehabilitation, while more recent work has shifted toward strain-defined probiotic supplementation. Likewise, gradual fiber reintroduction during refeeding should be distinguished from trials of isolated prebiotic supplementation.

Evidence on microbiome modulating interventions remains limited, even when considering eating disorders beyond AN. In a recent review of probiotic and prebiotic interventions for eating disorders, Baenas et al. screened 111 records and identified only five eligible studies, all in AN [87]. Another systematic review by Bahari et al. included 12 studies, but this broader evidence base combined animal and clinical studies and covered different eating-disorder presentations, intervention types, and outcomes [88]. For this reason, it was used to contextualize the field, whereas AN-specific human intervention studies were given greater weight for clinical interpretation. At present, the field lacks a coherent body of AN trials. Most studies are small, vary in patient age and treatment setting, and use non-interchangeable outcomes, including immune markers, body weight, constipation, appetite-related outcomes, mood, or microbiome composition.

Previous research on interventions for AN has not explored probiotics as a standalone treatment. Instead, it primarily examined fermented dairy products used during nutritional care. For instance, Solis et al. investigated fermented milk in malnourished children and AN patients undergoing nutritional recovery, noting increased interferon-γ production in AN patients who consumed yogurt [89]. Later, Nova et al. conducted a 10-week parallel nutritional intervention where 16 AN patients and 16 healthy adolescents consumed 375 g/day of yogurt containing *Lactobacillus bulgaricus* and *Streptococcus thermophilus*, while control groups consumed 400 mL/day of semi-skimmed milk [90]. The intervention was associated with changes in lymphocyte subsets and cytokine production capacity, including higher interferon-γ production in the yogurt group. These studies indicate that live-culture dairy products can influence immune-related outcomes during nutritional rehabilitation, but they do not show targeted modification of AN-associated gut microbiome alterations, improvement in eating-disorder psychopathology or sustained clinical recovery.

Trombetti et al. examined a different form of fermented dairy intervention. In their multicenter, randomized, double-blind trial, 62 adult women with AN and low baseline serum IGF-I received two daily isocaloric fresh-cheese portions for four weeks during inpatient refeeding. The higher-protein product provided 15 g protein per 150 g, compared with 3 g per 150 g in the control product [91]. Serum IGF-I increased in both groups, with a larger increase in the higher-protein group. However, the primary between-group analysis did not reach statistical significance. A post hoc mixed-regression analysis suggested greater increases in IGF-I and in the IGF-I/IGF-BP3 ratio, while bone turnover markers did not differ between groups [91]. These post hoc findings should be interpreted as exploratory. Because fecal microbiome, SCFAs and other microbial-metabolite outcomes were not measured, the trial is best interpreted as a fermented, protein-fortified dairy intervention during refeeding. It suggests that increasing protein intake through this food matrix may modestly improve IGF-I during nutritional rehabilitation, but it does not identify gut microbiome modulation as the mechanism.

The most directly applicable probiotic trial in AN is the randomized, double-blind, placebo-controlled study by Žaja et al. in children and adolescents with AN and constipation. Participants received *Limosilactobacillus reuteri* DSM 17938 or placebo during nutritional rehabilitation [92]. Constipation relief after three months was the primary endpoint, while secondary outcomes included stool frequency and body-weight normalization. The primary endpoint was constipation relief after three months, and superiority of the probiotic over placebo for this endpoint was not clearly demonstrated. More favorable bowel-movement patterns and body-weight normalization were reported as secondary outcomes. These secondary findings are clinically interesting, but they should not outweigh the non-significant primary endpoint. The possible role of this probiotic is therefore limited to symptom-targeted bowel support in pediatric AN patients with constipation, and the trial does not establish probiotics as routine treatment for AN, weight restoration or targeted microbiome modulation [92].

More rigorous AN-specific probiotic trials have been designed, but published efficacy evidence remains limited. Gröbner et al. published the protocol for a two-center, double-blind, randomized, placebo-controlled trial in adolescents with AN, testing a six-month multi-strain probiotic containing Lactobacilli and Bifidobacteria against placebo, with healthy controls followed in parallel [93]. Planned outcomes included microbiome composition, body mass index, gastrointestinal complaints, eating-disorder psychopathology, depression, anxiety, inflammatory markers, intestinal permeability, neuropsychological measures and neuroimaging endpoints [93]. The trial registry lists the study as completed, but no peer-reviewed efficacy publication or posted registry results could be identified at the time of writing. It should therefore be cited as evidence of trial development, not as evidence of probiotic efficacy in AN.

Overall, AN-specific probiotic evidence remains limited and heterogeneous. Favorable secondary or exploratory findings should not be interpreted as establishing clinical efficacy when the primary endpoint is not clearly positive. Current evidence therefore does not support routine probiotic, prebiotic or synbiotic use for weight restoration, eating-disorder psychopathology, mood symptoms, gastrointestinal symptom relief or targeted microbiome modulation in AN. Probiotics may be considered only as symptom-targeted adjuncts when a specific gastrointestinal indication is present, such as constipation or irregular bowel function, and when safety has been reviewed and their use does not interfere with nutritional rehabilitation. Their role should remain secondary to adequate intake, medical monitoring, bowel management and psychological treatment, and they should not be introduced in a manner that reinforces food-related fears or dependence on supplements.

### 7.2. Food-Based Strategies Relevant to the Gut Microbiome

Beyond probiotic-based interventions, a more common dietetic issue is how fiber-containing foods, fermented foods and broader dietary variety may be reintroduced during AN rehabilitation, without compromising energy adequacy, tolerance or flexibility. This should not be framed as a separate “microbiome diet”. In practice, the relevant variables are the food, portion size, timing, texture, energy contribution and tolerance. Human fiber-intervention studies outside AN show that effects on fecal SCFAs and gut microbiome composition vary according to fiber type, dose, duration and baseline microbiome [39,94]. This limits direct transfer to AN, but it is clinically useful because it separates gradual food-based fiber restoration from isolated prebiotic supplementation.

In clinical practice, fiber progression is best considered in relation to nutritional adequacy and gastrointestinal tolerance, rather than as a microbiome-specific target. Soluble, viscous or more fermentable fibers and insoluble, bulking fibers differ in their physiological effects, although whole foods usually contain mixed fiber types [94]. During early refeeding, rapid increases in high-volume fiber-rich foods or isolated fiber supplements may worsen fullness, bloating or abdominal distension, especially when postprandial sensations are already feared [64,69,95]. A pragmatic approach is to reintroduce fiber-containing foods after regular eating and adequate energy intake have been established, and then to expand them in small, repeated exposures according to stool pattern, hydration, abdominal symptoms and the patient’s ability to maintain adequate food intake. [8,64]. The practical target is not a high-fiber diet in the early phase of recovery, but a gradual return of tolerated fiber-containing foods without displacement of energy-dense foods. In AN, fiber should not be presented as a separate dietary target because patients may interpret it as another rule about permitted or preferred foods, whereas the aim is the gradual return of tolerated fiber-containing foods within a nutritionally adequate and varied diet [13].

Fermented foods require the same caution. The ISAPP consensus defines fermented foods as foods made through desired microbial growth and enzymatic conversion of food components, but fermented foods are not automatically probiotics unless they contain defined live microorganisms with demonstrated health benefits [38]. For AN rehabilitation, this distinction is practical because yogurt, kefir or other fermented foods may be acceptable components of dietary expansion, but they should not be presented as therapeutic substitutes for strain-defined probiotics. In healthy adults, Wastyk et al. showed that a diet high in fermented foods increased microbiome diversity and reduced several inflammatory markers, whereas a high-fiber diet produced different microbiome and immune effects [37]. The relevance for AN is indirect. Fermented foods may be considered as part of dietary expansion when they are acceptable and tolerated, but evidence from healthy adults does not establish efficacy during AN rehabilitation or justify their use as microbiome-specific therapy.

Dietary diversity may be more relevant to AN rehabilitation than isolated microbiome-directed ingredients. Large-cohort data outside AN link higher dietary diversity to differences in gut microbial composition, fecal metabolites, and host metabolic markers [96]. Polyphenol-rich foods also interact with the gut microbiome, since many polyphenols are metabolized by intestinal microbes and may in turn influence microbial composition [97]. However, evidence for polyphenols as prebiotic-like interventions remains stronger in preclinical than in human clinical studies, so this evidence should not be translated into a separate polyphenol prescription during AN recovery [98]. In AN, polyphenol-rich foods, fermented foods and fiber should not be treated as separate microbiome-directed tools. Their value depends on whether they help restore flexible dietary variety while maintaining energy adequacy. In recently weight-restored AN patients, lower dietary energy density and restricted dietary variety have been associated with poorer outcome, with lower dietary energy density also replicated as a relapse-related risk marker [44,99]. Fruit, vegetables, legumes, whole grains, nuts, seeds, cocoa, tea or other plant foods should not be introduced in a way that replaces energy-dense foods, reduces dietary fat, increases meal volume beyond tolerance or reinforces restrictive low-energy food choices, especially because restrictive food choice in AN often involves avoidance of higher-fat foods and may persist despite acute weight restoration [100,101].

Food-based strategies with relevance to the gut microbiome are best framed as pragmatic dietetic considerations within rehabilitation. They are appropriate when they support adequate intake, dietary variety, bowel regularity, gastrointestinal tolerance and reduced dietary rigidity. They are less appropriate when they create a separate microbiome-focused agenda, encourage supplement dependence, justify microbiome testing, target individual taxa or imply that recovery requires the restoration of gut microbiome.

## 8. The Role of the Dietitian in Microbiome-Informed Nutritional Rehabilitation

The previous sections suggest that gut microbiome research may broaden our understanding of nutritional rehabilitation in AN, potentially complementing but not replacing the main priorities of care. Weight restoration, medical stability, adequate energy and nutrient intake, gastrointestinal tolerance and dietary flexibility remain the clinical foundation. With current evidence, the question is not yet how to treat dysbiosis in AN, but how microbiome-related knowledge can help refine the monitoring and pacing of nutritional care. Reviews of gut microbiome in AN do not currently support routine microbiome testing or the use of microbiome profiles to guide dietary interventions in clinical practice [36,43]. Intervention studies of probiotics, prebiotics and other microbiome-directed approaches also remain few and heterogeneous, and current evidence is limited to justify routine use for AN [87,88]. Nutritional care should therefore remain based on individualized weight restoration goals, including target weight and expected weekly weight gain [102], medical risk monitoring [8,103] and dietary counseling delivered within coordinated multidisciplinary treatment [8]. Future research will be needed to determine whether more targeted microbiome-based interventions could eventually have a role in clinical practice.

As highlighted above, treatment of AN requires a multidisciplinary approach combining medical supervision, nutritional rehabilitation and evidence-based psychotherapy. Alterations in gut–brain interaction and gastrointestinal symptoms occur frequently in eating disorders and might contribute to anxiety, interoceptive distress, and restrictive eating behaviors [104]. In this context, brain–gut behavioral interventions (e.g., cognitive behavioral therapy) could be valuable for treating ongoing symptoms; however, present scientific literature does not justify their use in lieu of standard AN treatment or microbiome-directed intervention [36,70,105]. Moreover, restrictive symptom-directed diets, including the low-FODMAP diet, should generally be approached cautiously during nutritional rehabilitation due to the possibility of reducing the adequacy of energy intake, food variety, and increasing food avoidance. When clinically relevant gastrointestinal conditions are present, dietary interventions should be individualized, time-limited, and supervised by a dietitian or other clinical nutrition professional, experienced in eating-disorder patients [104].

**Figure 2:**
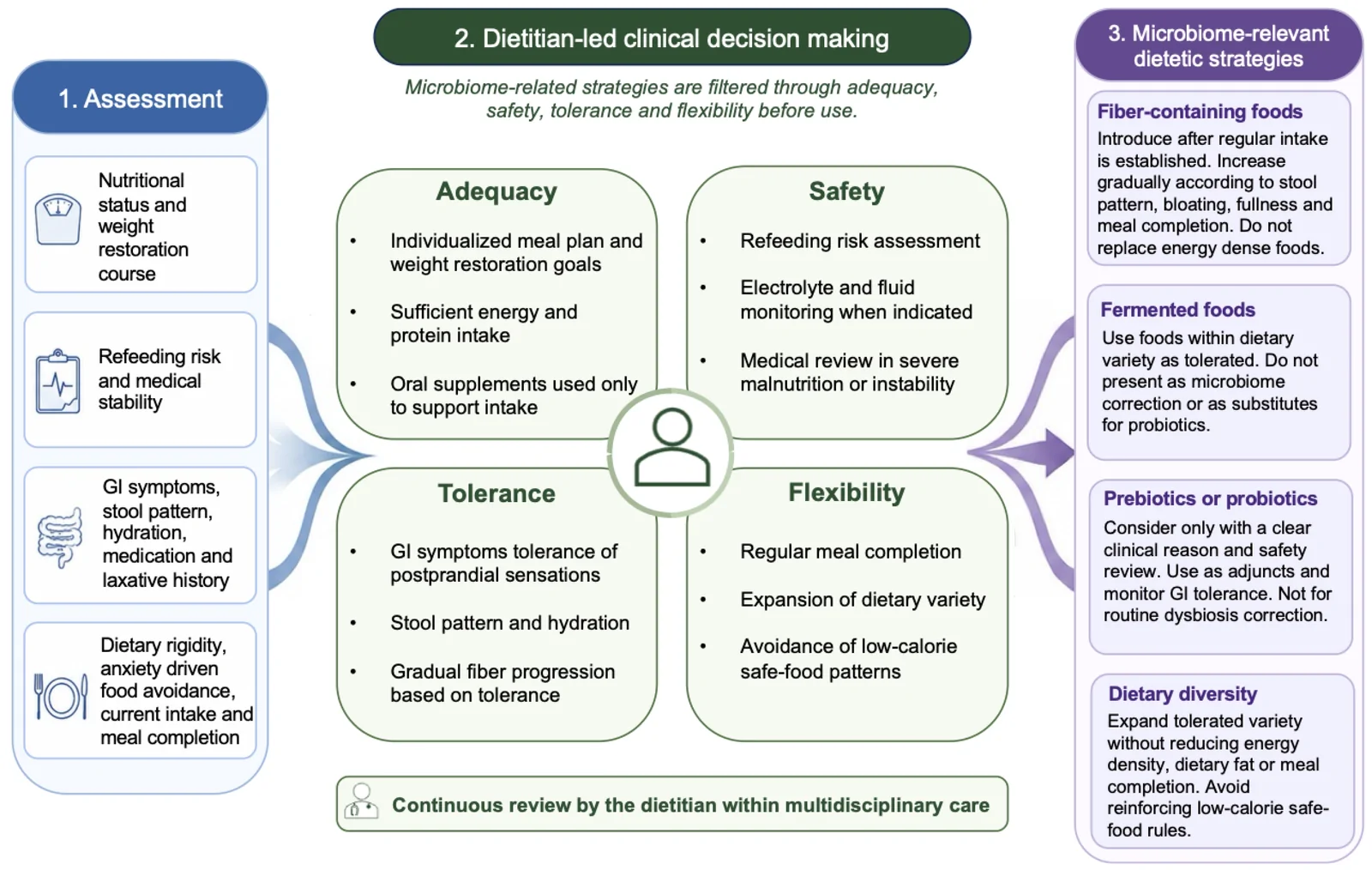
Proposed conceptual framework for microbiome-informed dietetic care during anorexia nervosa rehabilitation. Dietary options with relevance to the gut microbiome are considered only after nutritional adequacy, medical safety, gastrointestinal tolerance and dietary flexibility have been assessed. This figure is intended as a pragmatic framework, not as a validated clinical pathway or decision algorithm. It does not support routine microbiome testing, the use of microbiome profiles to guide dietary treatment, attempts to modify selected bacterial taxa or any intervention that compromises adequate intake or weight restoration. Abbreviations: GI, gastrointestinal.

In clinical practice, microbiome-related knowledge can be applied through the routine decisions that dietitians already make during AN rehabilitation. These include assessing whether energy and protein intake are sufficient, whether meals are completed and whether oral supplements support rather than replace food exposure [8,13,41]. Gastrointestinal symptoms, stool pattern, hydration and laxative or medication history can guide the pace of dietary progression [63,64,69]. At the same time, foods avoided because of anxiety about energy content, fullness, weight gain or loss of control, together with dietary rigidity and low-energy food choices, need to be considered because dietary variety and energy density are relevant to outcome after weight restoration [44,99]. Figure 2 presents a proposed conceptual framework for this process. It is intended as a pragmatic guide for organizing dietetic considerations during AN rehabilitation, not as a validated clinical decision algorithm.

In clinical practice, this means that microbiome-related strategies should remain rehabilitation centered. In early refeeding, regular and sufficient intake has priority, with appropriate monitoring for refeeding syndrome in patients at risk [67,103]. Once intake is more stable, gradual reintroduction of fiber-containing foods may be considered according to stool pattern, bloating, fullness and the patient’s ability to consume planned meals [63,64,69]. Fermented foods may be included as tolerated foods within dietary variety, but should not be presented as substitutes for strain-defined probiotics [85,86]. Probiotics or prebiotics are best reserved for situations with a clear clinical rationale and safety review, not as routine microbiome-directed treatment [87,88]. Dietary diversity should be expanded only when it supports energy adequacy, dietary fat and regular food intake, rather than reinforcing restrictive low-energy food choices [44,99].

At present, routine clinical management of AN does not require microbiome measurement. Established dietetic variables can be used to judge whether rehabilitation is restoring the dietary conditions that shape substrate availability for the gut microbiome. Clinical assessment should remain focused on adequate intake, meal completion, tolerance of dietary expansion, restoration of food variety and reduction in restrictive food rules, rather than on correction of individual bacterial groups.

Future studies should move beyond describing microbial differences before and after weight gain and investigate the clinical relevance of these changes. Longitudinal cohorts and trials should measure completed intake, diet composition, dietary variety, oral supplement use, fiber progression, gastrointestinal symptoms, stool pattern, laxative or medication exposure and treatment setting alongside microbiome and metabolite outcomes. This would help clarify whether microbial and fecal metabolite changes reflect weight gain itself, the type of intake achieved, time in recovery, bowel function or the degree of dietary expansion. Studies of probiotics, prebiotics, fermented foods or fiber should also define whether the aim is symptom relief, improved tolerance, microbiome change, metabolite change or support of dietary variety.

Finally, microbiome-focused dietary messages may inadvertently reinforce restrictive eating patterns. Recommendations that emphasize food exclusion, rigid “gut health” rules or selective consumption of specific foods may increase dietary rigidity and interfere with nutritional rehabilitation. Microbiome-informed strategies should therefore prioritize flexibility, adequacy and normalization of eating behaviors over the addition of new dietary restrictions.

## 9. Limitations

Several limitations should be acknowledged. Most studies of the gut microbiome in AN include small samples and differ in design, population, treatment setting and timing of assessment. Cross-sectional studies, short-term longitudinal cohorts, intervention studies and translational models therefore cannot be interpreted with the same level of clinical certainty. Differences in age, illness duration, illness stage and degree of weight restoration further limit comparisons across studies. In addition, most cohorts are predominantly or exclusively female, which restricts generalizability to males and more diverse AN populations.

Interpretation is further limited by methodological heterogeneity across microbiome and metabolite studies, including differences in sequencing methods, taxonomic resolution, sample handling, bioinformatic pipelines, metabolite assessment and reporting of functional pathways. Key clinical confounders are also not consistently measured or adjusted for. These include habitual and recent dietary intake, achieved energy intake during refeeding, oral supplement use, fiber intake, bowel transit, stool pattern, constipation, laxative use or withdrawal, medication exposure and physical activity. As a result, it remains difficult to determine whether observed microbiome and metabolite differences reflect AN itself, nutritional rehabilitation, bowel function, diet composition or time in recovery.

Finally, this is a narrative review and did not include formal risk-of-bias assessment or quantitative synthesis. The dietary and microbiome-related issues discussed here should therefore be interpreted as pragmatic considerations within established nutritional rehabilitation, rather than as microbiome-specific treatment recommendations.

## 10. Conclusions

Weight restoration remains the cornerstone of the treatment of AN, but weight gain may not coincide with improvement in gastrointestinal symptoms, reintroduction of a wider range of foods or changes in gut microbiome composition. Nutritional rehabilitation should therefore aim to achieve adequate energy and nutrient intake, regular consumption of meals, bowel regularity and the gradual reintroduction of a wider range of foods without worsening gastrointestinal symptoms or reinforcing restrictive eating. Changes in the gut microbiome may accompany these processes, but current findings do not establish a causal relationship between dysbiosis and AN or whether dysbiosis represents a clinically actionable therapeutic target. Studies evaluating the effects of probiotics, prebiotics, synbiotics and fermented foods during nutritional rehabilitation in people with AN remain few and heterogeneous. To date, available evidence is insufficient to recommend their routine use as strategies to enhance weight restoration, alleviate gastrointestinal symptoms, improve eating-disorder psychopathology or meaningfully modulate the gut microbiome. Fiber-containing foods, fermented foods and broader dietary variety may be included when they support nutritional adequacy and are tolerated without increasing meal volume excessively or displacing energy-dense foods. Within multidisciplinary treatment, dietitians can use information on actual intake, gastrointestinal tolerance, bowel function and restrictive food choices to guide nutritional rehabilitation. For the nutritional rehabilitation team, practical priorities include documenting achieved intake, monitoring gastrointestinal tolerance, stool pattern, hydration, bowel function, laxative or medication exposure and restrictive food choices, and advancing dietary variety in a way that supports flexibility rather than new food rules. Future studies should distinguish the effects of weight gain from those of diet composition, achieved intake, bowel transit, medication exposure and time in recovery, and determine whether microbiome changes are associated with sustained improvements in gastrointestinal tolerance, dietary variety and longer-term outcomes. Given the complexity of the gut ecosystem, research should also expand beyond the bacteriome, to better characterize the underexplored mycobiome, virome and archaeome and combine functional analyses of microbial activity and metabolite profiles.

## Figures and Tables

**Table 1:** Nutrition relevant alterations in the gut microbiome in anorexia nervosa and their interpretation during nutritional recovery.

Microbiome Feature	Findings Reported in AN	Main Supporting Evidence and Applicability	Relevance to Nutritional Recovery	Interpretation and Limitations
Microbial diversity	Lower alpha diversity has been reported in some cohorts, although findings vary by study and diversity index. Altered beta diversity may persist after short-term weight gain.	Review-level evidence and AN-specific human studies [20,29,46,47,48]	Microbial diversity can indicate differences between patients and controls, but it cannot by itself define gut-related recovery.	Findings vary by age, illness stage, sequencing method, treatment setting and statistical analysis.
Butyrate producing and carbohydrate fermenting bacteria	Several studies report lower or altered abundance of carbohydrate-fermenting and butyrate-associated taxa, including *Roseburia*, *Faecalibacterium*, *Lachnospira*, *Blautia* and *Agathobacter*.	AN-specific human studies, with human–translational evidence [16,17,18,47]	These taxa are relevant to the fermentation of non-digestible carbohydrates and SCFA production, especially butyrate.	The affected taxa differ across studies. Lower abundance does not prove causality or identify a single bacterial target for treatment.
Mucin-degrading bacteria	Increased or altered abundance of mucin-degrading bacteria, including *Akkermansia muciniphila*, has been reported in some AN studies and reviews.	AN-specific human studies and review-level evidence [17,30,43,49]	When dietary substrates are limited, greater use of host-derived mucus may reflect adaptation to a substrate-poor intestinal environment.	*Akkermansia muciniphila* should not be described as harmful by itself. Its interpretation depends on the nutritional state and the wider gut environment.
Short-chain and branched-chain fatty acids	Lower concentrations of selected fecal SCFAs have been reported in AN. Altered BCFA profiles have also been described after weight gain.	AN-specific human metabolite studies [17,18,19,45,47]	SCFAs and BCFAs help describe microbial fermentation and substrate use during restriction and refeeding.	The affected metabolites differ across studies, and fecal concentrations are influenced by production, absorption, stool transit, sample handling and recent intake. They are not standalone markers of microbiome-related recovery.
Neurotransmitter-related fecal metabolites	Lower fecal serotonin, GABA and dopamine have been reported in severe anorexia nervosa.	AN-specific human evidence; mainly severe AN [47]	These metabolites may indicate altered intestinal neuroactive metabolism during severe illness.	Fecal levels cannot be assumed to represent central neurotransmission and do not prove effects on appetite, mood or eating behavior.
Functional pathways	Shotgun metagenomic data suggest differences in microbial functions, including fermentation pathways and carbohydrate degradation capacity.	AN-specific human metagenomic evidence [50]	Functional data help explain how the gut microbiome may handle dietary substrates during restriction and refeeding.	Pathway findings need interpretation alongside dietary intake, metabolomics and clinical outcomes.
Gut barrier and immune findings	Links among dysbiosis, SCFAs, barrier function and immune signaling have been proposed. Recent adolescent data suggest immune dysregulation rather than a uniform inflammatory profile.	Mechanistic evidence, review-level evidence and AN adolescent data [31,42,51]	These pathways may help explain why gastrointestinal symptoms and food tolerance do not always improve in parallel with weight gain.	Human evidence is still limited. Cytokine findings differ according to age, illness phase, nutritional status and the marker studied.
Microbiome changes during recovery	Microbiome differences may persist after short-term weight gain, while longer follow-up data suggest partial movement toward healthy controls with recovery.	AN-specific longitudinal human studies and review-level evidence [17,29,45,46,47,48]	Microbiome-related changes during recovery may depend on time, energy intake, diet composition, hormonal recovery and the degree of weight restoration.	Short inpatient studies and one year follow-up studies represent different stages of recovery and should not be interpreted in the same way.

Abbreviations: AN, anorexia nervosa. SCFAs, short-chain fatty acids. BCFAs, branched-chain fatty acids.

**Table 2:** Representative human, review-level and translational evidence for microbiome-related pathways relevant to appetite, mood and recovery in anorexia nervosa.

Author and Year	Evidence Source/Design/Sample Size	Microbiome-Related Focus	Main Findings	Relevance
Prochazkova et al. 2021 [47]	AN-specific longitudinal human study; 52 patients with severe AN assessed before and after hospitalization/partial renourishment, compared with 67 healthy women controls	Fecal bacteriome, mycobiome, SCFAs and neuroactive fecal metabolites	Lower fecal acetate, butyrate, serotonin, GABA and dopamine were reported in severe AN. Metabolite and microbial profiles changed little after partial renourishment.	Supports altered intestinal metabolism during severe illness and shows that short-term renourishment may not restore fecal metabolite profiles.
Monteleone et al. 2021 [45]	AN-specific human multi-omics study; 21 women with AN in the underweight phase, 16 after short-term weight restoration, and 20 healthy women controls	Fecal microbiome and metabolite relationships	Reduced bacterial richness and a higher *Bacteroidota*/*Bacillota* ratio (reported as *Bacteroidetes*/*Firmicutes* ratio in the original study) persisted after short-term weight restoration, alongside altered bacteria–metabolite correlations.	Supports the idea that weight restoration alone does not necessarily restore microbiome–metabolite relationships.
Käver et al. 2024 [51]	AN-specific longitudinal adolescent human study; 63 female adolescents with AN at admission, 50 at discharge, 45 at 1-year follow-up, and 41 healthy female controls	Cytokines and gut microbiome	IL-1β and IL-6 were lower during the acute phase, IL-1β normalized after weight recovery, TNF-α did not differ significantly and IL-15 remained elevated. Cytokine–microbiome associations were also reported.	Supports immune dysregulation during illness and recovery, but not a uniform inflammatory profile.
Dalton et al. 2018 [75]	Evidence synthesis; meta-analysis of cytokine concentrations in eating disorders, including 25 studies, 632 participants with eating disorders and 487 healthy controls	Systemic inflammatory markers	Altered cytokine concentrations were reported across eating disorders, including AN.	Provides broader support for immune involvement, while requiring careful interpretation because studies differ in design and patient status.
Solmi et al. 2015 [74]	Evidence synthesis; meta-analysis of cross-sectional and longitudinal cytokine studies in AN, including 22 studies and 924 participants	Cytokines in AN	Inflammatory markers differed in AN, although findings varied across cytokines and study designs.	Supports the inclusion of immune signaling as a possible pathway, without treating inflammation as a single mechanism.
Keeler et al. 2025 [76]	Evidence synthesis; updated systematic review and meta-analysis of cytokines in eating-disorder populations, including 53 studies overall and 43 studies in meta-analyses	Cytokines in eating-disorder populations	Higher IL-6 and IL-15 and lower IL-7 were reported in AN, while several other cytokines did not differ after sensitivity analyses.	Adds current evidence that immune findings are heterogeneous rather than uniformly pro-inflammatory.
Tennoune et al. 2014 [35]	Experimental study with human eating-disorder antibody data; mouse immunization and bacterial-gavage experiments, with plasma antibody data from patients with AN, BN and BED	ClpB and α-MSH molecular mimicry	Bacterial ClpB was identified as a conformational mimetic of α-MSH. ClpB immunization induced cross-reactive antibodies and affected food intake, body weight, anxiety-related behavior and melanocortin signaling in mice.	Provides one of the most specific proposed microbiome–appetite pathways in eating disorders.
Lucas et al. 2019 [77]	Human eating-disorder and obesity study with experimental MC4R-signaling assays; female participants included AN, BN, BED, obesity and healthy control groups	α-MSH-reactive IgG and MC4R signaling	α-MSH-reactive IgG were increased in anorectic and bulimic patients, and α-MSH/IgG immune complexes interacted with MC4R signaling.	Strengthens the immune–melanocortin component of the ClpB/α-MSH hypothesis.
Fetissov and Hökfelt 2019 [78]	Mechanistic review; sample size not applicable	Gut microbiome, adaptive immunity and melanocortin signaling	Altered signaling between gut microbiome, adaptive immunity and melanocortin pathways was proposed as relevant to eating disorders.	Places ClpB/α-MSH within a wider microbiome–immunity–satiety hypothesis.
Bremard et al. 2025 [79]	Exploratory AN-specific human fecal proteomic study; fecal samples from 6 patients with AN and 6 healthy controls	α-MSH-like enterobacterial proteins	α-MSH-like enterobacterial proteins, including ClpB, were detected in fecal microbiome samples from patients with AN.	Provides emerging human evidence, but the small exploratory design limits clinical interpretation.
Fan et al. 2023 [16]	AN-specific human multi-omics study with translational FMT experiment; 77 female AN cases and 70 healthy female controls, with AN-derived microbiota transferred to germ-free mice	AN-associated microbiome, metabolites and host energy regulation	AN was associated with altered bacterial taxa, viral components, functional modules and serum metabolites. Mice receiving AN microbiome under energy restriction gained less weight and showed changes in hypothalamic and adipose gene expression.	Provides translational evidence that AN-associated microbiome can affect host metabolism under experimental conditions.
Hata et al. 2019 [80]	Translational animal model using microbiota from patients with AN; fecal samples from 4 restricting-type AN patients and 4 healthy controls were transferred to female germ-free mice	Weight gain and behavioral performance	AN-derived microbiome impaired weight gain and behavioral performance in female mice.	Supports a possible transferable effect of AN-associated microbiome on host phenotype.
Glenny et al. 2021 [81]	Translational animal model using human AN and non-AN donor microbiota; 4 AN donors sampled before and after renourishment, 4 non-AN donors, and 153 recipient germ-free mice	Body weight outcomes	Gut microbial communities from patients with AN did not influence body weight in recipient germ-free mice.	Adds important contradictory evidence and prevents overstatement of causality.
Kooij et al. 2024 [82]	Translational animal model using FMT from adolescent AN and healthy-control donors to rats; 46 TH-cre Long Evans rats initially used, with fecal material from 3 AN donors and 3 healthy-control donors	Cognitive flexibility, anxiety and dopamine-related endpoints	FMT from patients with AN did not alter flexible behavior in rats.	Shows that microbiome-transfer effects may depend on model, endpoint and engraftment.
Maschek et al. 2025 [83]	Translational animal model using FMT from human AN and control donors in antibiotic-treated mice; 48 mice, with FMT from 3 AN donors and 3 control donors	Food intake, appetite-related hormones and gut microbiome	FMT from AN donors was associated with lower food intake and changes in gut microbiome and appetite-related hormones, but without a corresponding body-weight effect in the first transplant phase.	Supports interest in appetite-related signaling, while showing that effects are endpoint-specific and not always reflected in body weight.
Garcia and Gutierrez 2023 [70]	Evidence synthesis; systematic review and critical appraisal of AN microbiota evidence, including 18 human and animal studies	AN and gut microbiome evidence	The review emphasized heterogeneity of findings and the need for caution when interpreting microbiome differences in AN.	Microbiome-related pathways are plausible, but clinical causality remains unproven.

Abbreviations: AN, anorexia nervosa. FMT, fecal microbiome transplantation. GABA, γ-aminobutyric acid. IgG, immunoglobulin G. MC4R, melanocortin-4 receptor. SCFAs, short-chain fatty acids. α-MSH, α-melanocyte-stimulating hormone. ClpB, caseinolytic peptidase B.

## Data Availability

No new data were created or analyzed in this study. Data sharing is not applicable to this article.

## Abbreviations

The following abbreviations are used in this manuscript:

AΝ	anorexia nervosa
BCFA	branched-chain fatty acid
BMI	body mass index
ClpB	caseinolytic peptidase B
FMT	fecal microbiota transplantation
GABA	gamma-aminobutyric acid
GI	gastrointestinal
IGF-I	insulin-like growth factor I
IGFBP-3	insulin-like growth factor-binding protein 3
IgG	immunoglobulin G
IL-1β	interleukin-1 beta
IL-6	interleukin-6
IL-7	interleukin-7
IL-15	interleukin-15
ISAPP	International Scientific Association for Probiotics and Prebiotics
MC4R	melanocortin-4 receptor
PYY	peptide YY
SCFA	short-chain fatty acid
StRONG	Study of Refeeding to Optimize Inpatient Gains
TNF-α	tumor necrosis factor alpha
α-MSH	alpha-melanocyte-stimulating hormone
